# AGE-FRIENDLY CITIES AND COMMUNITIES: RESEARCH ON LOCAL PRACTICE TO STRENGTHEN THE MOVEMENT’S FUTURE

**DOI:** 10.1093/geroni/igad104.0334

**Published:** 2023-12-21

**Authors:** Emily Greenfield, Natalie Pope, Althea Pestine-Stevens

**Affiliations:** Rutgers, The State University of New Jersey, New Brunswick, New Jersey, United States; Rutgers Social Work, New Brunswick, New Jersey, United States; Rutgers, The State University of New Jersey, New Brunswick, New Jersey, United States

## Abstract

The age-friendly cities and communities (AFCC) movement has accelerated a major paradigmatic shift for the field of aging in the 21st century. Launched and spurred by high-profile government agencies and organizations across the globe, this ambitious movement highlights the transformative potential of deliberate, cross-sectoral efforts to improve built, social, and service environments of localities in response to population aging. This presentation will provide a brief overview of the development of a global program of policy and practice on AFCCs, with an emphasis on its emergence in the United States. It will then present insights regarding challenges and opportunities for the future based on findings from an ongoing, longitudinal study of a regional network of AFCC initiatives. The study, designed as a developmental evaluation, explores the perspectives of on-the-ground AFCC leaders on their practice as part of a private philanthropic grantmaking initiative to spur AFCCs in New Jersey. Reflecting on our years of engaged research with this network, the presentation will highlight issues concerning multi-level systems alignment, the promises and pitfalls of public sector leadership, the need for additional practice theories, and greater incorporation of considerations of aging equity and racial justice. The presentation will demonstrate the importance of continuing to leverage the strengths of community-engaged research to better ensure the movement’s relevance, reach, and equitable impact across diverse community, organizational, and broader systems contexts.

